# Role of IgG4 Antibodies in Human Health and Disease

**DOI:** 10.3390/cells14090639

**Published:** 2025-04-25

**Authors:** Li-li Shi, Peng Xiong, Minglin Yang, Ozge Ardicli, Stephan Raphael Schneider, Anders Boutrup Funch, Ayca Kiykim, Juan Lopez, Cezmi A. Akdis, Mübeccel Akdis

**Affiliations:** 1 Swiss Institute of Allergy and Asthma Research (SIAF), University of Zurich, 7265 Davos, Switzerland; shilili.tj@163.com (L.-l.S.); xp3345@163.com (P.X.); ozgeyilmaz@uludag.edu.tr (O.A.); stephanr.schneider@siaf.uzh.ch (S.R.S.); abfunch@sund.ku.dk (A.B.F.); ayca.kiykim@iuc.edu.tr (A.K.); juan.lopez@siaf.uzh.ch (J.L.); cezmi.akdis@siaf.uzh.ch (C.A.A.); 2 Department of Otolaryngology-Head and Neck Surgery, Tongji Hospital, Tongji Medical College, Huazhong University of Science and Technology, Wuhan 430030, China; 3 Department of Pediatrics, Tongji Hospital, Tongji Medical College, Huazhong University of Science and Technology, Wuhan 430030, China; 4 Division of Food Processing, Milk and Dairy Products Technology Program, Karacabey Vocational School, Bursa Uludag University, Bursa 16700, Turkey; 5 LEO Foundation Skin Immunology Research Center, Department of Immunology and Microbiology, Faculty of Health and Medical Sciences, University of Copenhagen, 2200 Copenhagen, Denmark; 6 Department of Pediatrics, Division of Pediatric Allergy and Immunology, Cerrahpasa Medical School, Istanbul University-Cerrahpasa, Istanbul 34098, Turkey

**Keywords:** IgG4, immune tolerance, allergen-specific immunotherapy (AIT), autoimmune disease, allergic disease, chronic infection, tumor

## Abstract

Immunoglobulin G4 (IgG4), a unique subclass of IgG antibodies, plays diverse roles in human health and disease. Its distinct features, such as Fab-arm exchange and specific mutations, confer reduced effector functions compared to other IgG subclasses. In health, IgG4 responses contribute to immune tolerance, particularly in the context of allergen-specific immunotherapy (AIT), where they can mediate tolerance to environmental antigens, inhibit IgE-dependent mast cell degranulation, and compete with IgE for allergen binding. This helps in attenuating allergic symptoms and is associated with increased levels of allergen-specific IgG4. However, in disease scenarios, the role of IgG4 is complex. IgG4 lacks complement fixation and, thus, shows a reduced ability to activate immune effector pathways, it was initially thought to be protective against autoimmune diseases. However, emerging evidence suggests that it can contribute to pathology. For instance, IgG4 autoantibodies against specific antigens can aggravate conditions in certain autoimmune disorders. In some cancers, it may play a role in immune evasion, with higher levels correlating with poor patient survival, albeit in others, its exact function remains elusive. Overall, understanding the precise role of IgG4 in various physiological and pathological conditions is crucial for developing targeted therapeutic strategies and improving patient outcomes.

## 1. Introduction

Immunoglobulin gamma 4 (IgG4) antibodies are attracting attention within the field of immunology. Over the past few decades, research on IgG4 has significantly expanded our understanding of its diverse functions and implications in human health and disease.

IgG4 was at first considered a minor and relatively unimportant subclass of immunoglobulin, as it only constitutes approximately 5% of total serum IgG [1]. However, with advancements in immunological techniques and a growing body of clinical and experimental studies, it has become evident that IgG4 plays a complex and multifaceted role in humans but not in mice, as IgG4 does not exist in mice [2]. Although IgG4 responses play a protective role by mediating immune tolerance to environmental antigens and reducing inflammation during parasitic infections, they can also contribute to pathology in autoimmune diseases and antitumor settings [3,4,5]. Evolving as a mechanism to mitigate tissue damage and adapt to chronic exposure to high-dose antigens, IgG4 functions as a paramount component in the immune system’s balance between tolerance and overactivation [6,7]. In health, IgG4 is involved in maintaining immune homeostasis [8,9]. It is thought to participate in immune tolerance mechanisms [6], potentially modulating the immune response to self-antigens and harmless environmental antigens. For example, in the context of mucosal immunity, IgG4 may contribute to the regulation of responses against commensal bacteria and dietary antigens, preventing excessive inflammation and tissue damage [10,11].

In contrast, elevated IgG4 levels and their functionality have been associated with a wide range of diseases. In some autoimmune disorders, such as IgG4-related diseases (IgG4-RD), there is a characteristic increase in IgG4-producing plasma cells, and elevated serum IgG4 concentrations are observed [12]. While in isolated IgG4-related sclerosing cholangitis, the serum IgG4 levels are normal [13]. IgG4-RD can affect multiple organs, including the pancreas, salivary glands, and lymph nodes, leading to chronic inflammation and tissue fibrosis [14]. The exact pathophysiological mechanisms underlying IgG4-RD are still being elucidated, but it is clear that IgG4 antibodies are central drivers of the pathology.

Furthermore, in allergic diseases, the role of IgG4 is highly debated. While some studies suggest that IgG4 may act as a blocking antibody, inhibiting mast cell degranulation and thereby potentially protecting against allergic reactions, other evidence points to more complex and sometimes detrimental effects. For instance, in certain allergic conditions such as severe eosinophilic chronic rhinosinusitis complicated with asthma, the presence of high levels of IgG4 may be associated with persistent or severe disease manifestations [15,16].

Moreover, recent studies have also explored the potential role of IgG4 in cancer immunology [17]. There is growing interest in understanding whether IgG4 antibodies promote or suppress tumor growth and metastasis, as well as how they interact with other components of the immune system within the tumor microenvironment.

This review aims to comprehensively summarize the current knowledge regarding the role of IgG4 antibodies in human health and disease. By integrating findings from basic immunology research and clinical studies, we hope to provide a better understanding of the significance of IgG4 and its potential as a biomarker and therapeutic target in various pathological conditions.

## 2. The Structure of IgG4

**Figure 1:**
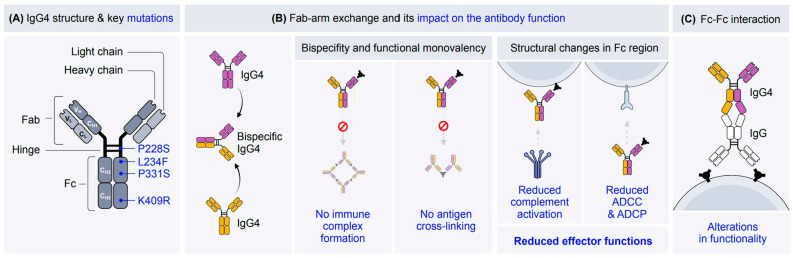
Schematic representation of IgG4 functional characteristics and mechanisms of action.

Assessment of IgG4’s evolutionary dynamics provides valuable insights into its role in immune regulation, pathogen interactions, various diseases, and malignancies [6]. The IgG4 molecule consists of two heavy chains and two light chains, each comprising variable (VH, VL) and constant (CH1, CH2, CH3, CL) domains. The hinge region connects the Fab and Fc regions, providing structural flexibility [18]. The unique structure of IgG4 is key to exerting its protective and pathogenic functions. Uniquely, IgG4 undergoes a process termed Fab-arm exchange that produces bispecific antibodies by recombining half-antibodies from two different IgG4 molecules. This creates functionally monovalent antibodies with reduced avidity for multivalent antigens, which contribute to their anti-inflammatory properties. The Fab-arm exchange mechanism is thought to be driven by a serine-proline switch in the hinge region that weakens the strength of the disulfide bond between the heavy chains and facilitates its dissociation into half-antibodies for further recombination with other IgG4 half-antibodies, resulting in a bispecific antibody [7,12]. IgG4’s unique structural features in the hinge region, CH2 domain, and CH3 domain form the basis of its distinct binding characteristics and reduced effector function. Compared to other IgG subclasses, IgG4 has a shorter and more flexible hinge region (12 amino acids), facilitating Fab-arm exchange [17,19,20] (Table 1). The flexibility of the hinge region affects its antigen-binding capacity, immune complex formation, and the accessibility of binding sites for C1q and/or Fc receptors, which may be partially or completely obscured by the Fab arms [18]. Certain key mutations confer upon IgG4 its unique structure; see Figure 1. In this context, the P228S mutation in the hinge region enables Fab-arm exchange and culminates in bispecificity. The critical functional features, including the Fcγ receptor binding and antibody-dependent cellular cytotoxicity (ADCC), are altered by the L234F mutation in the CH2 domain.

The IgG4 molecule is composed of two heavy chains and two light chains, namely, the variable (V) and constant (C) domains of the heavy chains (VH, CH1, CH2, and CH3) and the light chains (VL, CL). The hinge region connects the Fab and Fc regions and provides flexibility. Some key mutations enable IgG4 to exhibit specific characteristic properties. The P228S mutation in the hinge region contributes to structural flexibility and enables Fab-arm exchange, which leads to bispecificity. The L234F mutation in the CH2 domain of the Fc region influences binding to Fcγ receptors, altering effector functions such as antibody-dependent cellular cytotoxicity (ADCC). Another mutation in the CH2 domain (P331S) affects the binding of complement component C1q, reducing complement activation. The K409R mutation in the CH3 domain modifies interactions with Fcγ receptors and other immune effectors. (B) IgG4 antibodies are characterized by a phenomenon known as Fab-arm exchange, where the half-molecules of two different IgG4 antibodies swap, resulting in a bispecific antibody. This structural feature enables 99% of IgG4 antibodies to exhibit bispecificity. The ability to bind two different antigens through its distinct Fab regions is a key hallmark of IgG4 functionality. Due to Fab-arm exchange, IgG4 antibodies exhibit functional monovalency, which prevents simultaneous binding of the same antigen on different epitopes. This feature limits their ability to form large immune complexes and reduces immune activation. The Fc region of IgG4 undergoes structural changes that impair its interaction with Fc receptors and complement proteins. Consequently, IgG4 antibodies exhibit reduced effector functions, such as complement activation, ADCC, and antibody-dependent cellular phagocytosis (ADCP). The inability of IgG4 antibodies to cross-link antigens arises from their bispecific nature and functional monovalency, which reduce their capacity to mediate immune activation or phagocytosis. IgG4 antibodies are associated with immune tolerance mechanisms, particularly in chronic antigen exposure or allergen-specific immunotherapy. They are involved in blocking immune complex formation and reducing immune activation. In contrast, other IgG subclasses (IgGs) can form stable immune complexes. (C) IgG4 can interact with other IgGs, resulting in alterations in the functionality of the molecule. This mechanism likely involves the dissociation of the CH3 domains in IgG4 antibodies.

Additionally, the P331S mutation diminishes C1q binding, the first component of the classical complement activation pathway, thereby impairing complement activation. C1q is crucial for initiating the classical complement cascade. In the CH3 domain, the K409R mutation further modifies interactions with Fcγ receptors, contributing to IgG4’s overall reduced effector functions [7,12]. Another defining feature of IgG4 is the Fc-Fc interaction. This structural modification describes the interaction between human IgG4 and several other IgG subclasses. Furthermore, the potential for Fc–Fc binding with other IgG subclasses suggests that IgG4 may function as a scavenger, targeting IgG molecules with compromised structural integrity [17,28]. However, relatively scant attention has been directed toward the Fc–Fc interactions between IgG4 and other IgG Fc fragments, which may potentially give rise to adverse effects. A very recent study has revealed that IgG4 and nivolumab bind to immobilized IgG via Fc–Fc interactions. This binding phenomenon leads to a reduction in antibody-dependent cell-mediated cytotoxicity as well as phagocytosis reactions, showing the poor functioning of IgG4 in anti-PD-1 immunotherapies [29].

Taken altogether, these characteristics set IgG4 apart within the antibody family and define its unique role in immunity. The combination of various tolerogenic features not only minimizes its effector functions but also allows IgG4 to mediate immune regulation effectively. The unique structure of IgG4 provides insights into its function in a range of disease states, including autoimmune disorders, allergies, and tumor immunity, where balancing immune activation and suppression is critical. The specialized structure and function of IgG4 exemplify an evolutionary adaptation, enabling the immune system to protect the human body against foreign threats while maintaining tolerance and preventing excessive inflammation toward innocuous substances and self-proteins.

## 3. Protective Role of IgG4 in Human Health

IgG4 plays a pivotal role in both the allergic responses and the development of natural tolerance to antigens due to its inability to activate complement pathways, its bispecificity, and its monovalent nature, which prevent it from cross-linking antigens [30]. The type 2 response and allergen-epitope recognition are characteristic of both IgE and IgG4 antibodies [31,32,33]. However, it has been shown that in addition to type 2 cytokines, IgG4 induction mainly requires IL-10 [34,35]. In vivo models of high-dose allergen exposure and acquired natural tolerance (an outgrowth of allergic response without dose-controlled intervention) to allergens have shown increased titers of IgG4 [27].

A similar antigen-specific B cell response to bee venom exposure in both allergic patients after AIT and beekeepers has been demonstrated, with the latter serving as a model of natural tolerance [8,9,36]. Tolerant beekeepers who are exposed to high doses of bee venom elicited higher levels of functional blocking PLA2-specific IgG4 than allergic subjects who receive allergen immunotherapy [37,38]. Additionally, these subjects presented an enriched population of PLA2-specific IL-10-producing regulatory B cells expressing IgG4 [39].

Helminth infections are known to induce a strong type 2 response, but IL-10 has been shown to be necessary for infection persistence and host survival [40]. The presence of IL-10 promotes the induction of IgG4. This antigen-specific IgG4 protects the host from developing an uncontrolled type 2 response against the allergens of the parasites and contributes to the chronicity of the infection [7].

Animals are a major source of airborne allergens. Regular or prolonged exposure to cats and dogs may promote tolerance and may be protective against the development of asthma [41], marked by increased levels of IgG4 [42,43]. In the occupational field, high titers of rat urinary allergen-specific IgG4 have been found in laboratory animal workers [44]. However, its protective effect on developing sensitization or allergy is not clear [45,46,47]. In some studies, high serum levels of antigen-specific IgG or IgG4 may have been markers for clinical tolerance among laboratory animal workers with detectable allergen-specific IgE [46,47]. But another study showed that high levels of IgG4 showed no protective effect, as specific IgG4 antibodies were present before the development of allergies to laboratory animals and did not significantly change over time [45].

Children allergic to certain foods commonly develop natural tolerance; in other words, they outgrow the food allergy without any treatment. The underlying molecular mechanisms are still not fully understood. IgE and IgG4 epitope overlap might be a fundamental aspect in achieving natural tolerance to cow’s milk allergy [48]. In addition, milk allergen Bos d 9-specific B cells from children who naturally resolved their allergies produced higher levels of IgG4 compared to children before and after oral immunotherapy [11].

## 4. Protective Role of IgG4 in Allergen-Specific Immunotherapy

AIT is a disease-modifying treatment for IgE-mediated allergic diseases, inducing immune tolerance to allergens and providing long-term attenuation of symptoms. AIT is mediated by multiple protective mechanisms, including increasing IgG4 production and suppressing the secretion of IgE [49]. Even though human IgG4 is the least abundant IgG isotype, after AIT, B cells skew from predominantly IgE production to IgG4, a key antibody for achieving immune tolerance [50]. Allergen-specific IgG4 responses have been linked to a wide variety of AIT modalities, including subcutaneous immunotherapy (SCIT), sublingual immunotherapy (SLIT), and oral immunotherapy (OIT). These responses are observed in cases of allergen exposure, such as grass pollen, house dust mites, bee venom, and peanuts. In a study of AIT for grass pollen, IgG4 levels increased 100-fold after 19 months of therapy [51]. A two-year AIT study against grass pollen demonstrated higher clinical efficacy in the second year, which was accompanied by continuously increasing allergen-specific IgG4 [52]. AIT can successfully treat IgE-mediated allergies, often linked to the production of allergen-specific IgG4. But it is unclear if IgG4 prevents allergic reactions better than other IgG subclasses. A recent study compared allergen-specific monoclonal IgG1 and IgG4 antibodies’ ability to inhibit type I allergic reactions via FcγRIIb binding, and found that virus-induced IgG1 binds to FcγRIIb with similar affinity to IgG4 and is equally effective in blocking basophil activation in allergic patients by neutralizing allergens and binding to FcγRIIb. So, they reported that the influence of the IgG subclass type on the protective effect of AIT was limited. Even though IgG4 is regarded as the best indicator of protection, it is probably because classical AITs predominantly induce the production of IgG4 [53]. However, another study reported that the blocking activity of allergen-specific IgG1 should not be underestimated, particularly early in AIT, as both IgG1 and IgG4, as dominant IgE-blocking antibodies, shift during allergen immunotherapy [54].

**Figure 2:**
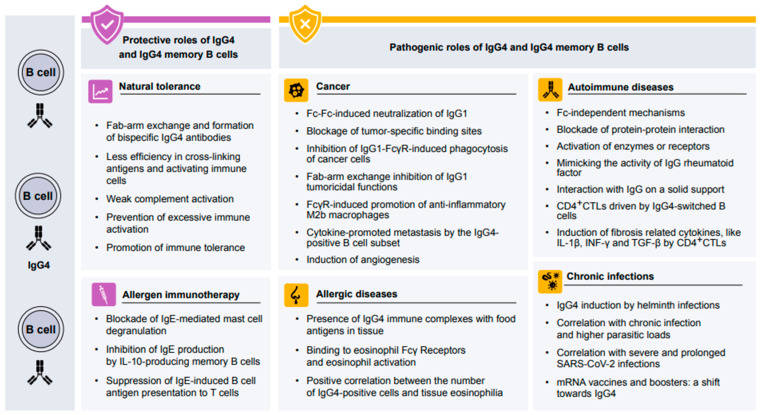
The mechanism of IgG4 in immune regulation in humans—IgG4 antibodies are a double-edged sword in human health and disease. IgG4 plays a protective role in natural tolerance and allergen-specific immunotherapy. They also play a pathogenic role in allergic diseases, autoimmune diseases, cancer, and chronic infections.

IgG4 and IgE production both rely on the Th2 cytokines IL-4 and IL-13. However, IL-10 produced by Treg and Breg cells might promote IgG4 secretion instead of IgE [55]. The ε transcript encoding the constant region of the IgE heavy chain and IL-4-induced IgE secretion are both inhibited by the addition of IL-10 under in vitro conditions [34]. The same study also confirms the promotional effect of IL-10 in producing gamma 4 transcripts, required for IgG4 heavy chain encoding and IgG4 production [34]. The IgG4 bispecificity originates from the Fab-arm exchange. This process confers upon IgG4 an anti-inflammatory and protective role as its functionally monomeric structure limits its ability to crosslink with allergens [3]. The IgG4 monovalency prevents the formation of immune complexes containing other antibody isotypes and, thus, it is only a weak activator of the complement system [56]. In AIT, IgG4 acts as an IgE inhibitor by competing with IgE for allergen binding and blocking IgE-dependent mast cell degranulation by binding to the inhibitory receptor FcγRIIb on mast cells [27]. Food allergen-specific IgG antibodies induced by AIT were initially found to mediate the inhibition of mast cell activation by FcγRIIb, accompanied by an increased level of food-specific IgG4 [57]. Plasma from peanut-sensitized but tolerant donors significantly suppressed the mast cell and basophil activation from peanut-allergic patients with OIT, and the inhibition was abolished after the depletion of IgG4 [58]. In addition, the allergen presentation to T cells induced by IgE, a key factor of allergen-specific Th2 responses, is also blunted by IgG4 blocking the IgE-receptor FcεRII on B cells after SLIT [59,60]. During AIT, both the quantity of allergen-specific IgG4 as well as the repertoire diversity have changed. A high-throughput peptide microarray system was used to quantify the increase in peanut-specific IgG4 after 41 months of immunotherapy and to evaluate the changes in specificity and diversity. [61]. The mechanisms operative in AIT are summarized in Figure 2, with an emphasis on the role of IgG4. The high total IgG4 amount was once associated with the failure of AIT in 1987, but R. Djurup and H.J. Malling compared three AIT studies, which used diverse antigens, administration methods, and strategies measuring IgG4; therefore, the conclusion should be reconsidered [62].

Identifying reliable biomarkers to predict the clinical outcome of AIT is still a challenge. The potential of IgG4 as a prognostic marker in AIT remains a subject of debate. Specific IgG4 levels against house dust mites did not correlate with AIT-combined symptom medication scores (CSMSs) in allergic rhinitis patients with or without asthma [63]. On the other hand, a study by a different group demonstrated a significant association between allergen-specific IgG4 and CSMS in AIT against house dust mites [64]. In addition, elevated IgG4 levels isolated from saliva during house dust mite AIT were positively correlated with treatment efficacy. The non-invasive approach of using salivary IgG4 as a biomarker could improve patient adherence to treatment [65]. All three studies evaluated the association between elevated IgG4 levels and CSMS during AIT but did not follow up after completion of AIT. Furthermore, preclinical studies showed that recombinant, allergen-specific blocking IgG4 antibodies function similarly to allergen-specific IgG from patients who had successful AIT. In cat-allergic patients during nasal allergen provocation, this leads to a quick and lasting decrease in clinical symptoms. Thus, it shows that allergen-specific blocking IgG is a key part of AIT’s protective mechanism and could be a new, faster allergy treatment [66]. Therefore, further research is warranted to confirm IgG4 as a long-term biomarker of tolerance, as an indicator of successful AIT outcomes, or as a potential therapeutic approach for patients with asthma and/or allergic rhinitis caused by house dust mites.

For food allergies, IgG4 is not supported as a diagnostic tool for immunotherapy by both the European Academy of Allergy and Clinical Immunology (EAACI) and the American Academy of Allergy and Immunology (AAAI) [67,68]. In addition, patients with AIT showed increased levels of peanut-specific IgG, but the level of peanut-specific IgG4 in saliva after immunotherapy was not higher in remission compared to the non-remission [69]. However, in a recent study on peanut allergy, it was observed that after immunotherapy, only patients who had a sustained clinical effect (as opposed to those with a transient effect) showed an increase in neutralizing IgG4 antibodies. These high-affinity IgG4 antibodies were found to block the peanut Ara h 2 epitopes, and the findings of the study support IgG4 as a potential biomarker in allergen immunotherapy [70]. In an EAACI position paper, IgG4 was suggested as a potential biomarker of clinical efficiency of AIT for allergic rhinoconjunctivitis and allergic asthma [71]. During AIT for Hymenoptera venom, clinical efficacy was associated with elevated IgG4 levels, as they confer protection against re-stings in patients with clonal mast cell disorders [72]. In atopic dermatitis, patients sensitized to house dust mites who underwent AIT exhibited increased allergen-specific IgG4 levels after 2 years of immunotherapy. This increase was associated with positive treatment outcomes, highlighting its potential as a biomarker of AIT [73]. These contradictory studies draw attention to our limited knowledge of the use of IgG4 as a biomarker of AIT clinical efficacy.

## 5. Pathogenic Role of IgG4 in Human Diseases

### 5.1. Pathogenic Role of IgG4 in Allergic Diseases

IgG4 is often elevated in the context of allergic disease and was thought to play a protective role by acting as a blocking antibody and inhibiting mast cell degranulation, but its role in disease pathogenesis is less clear [74]. In recent decades, there has been mounting evidence that also points to a pathogenic role for IgG4 in allergic diseases. For example, IgG4 was found to form immune complexes with food antigens that deposit in the esophageal tissue of patients with active eosinophilic esophagitis (EoE) [75], which is a form of food allergy and known to be characterized by a type 2 inflammatory response [76]. These immune complexes promote inflammation by binding to eosinophil Fcγ receptors, inducing eosinophil activation and degranulation [77]. Moreover, children with EoE have significantly higher serum levels of IgG4 to milk-specific proteins than unselected controls [78]. As Fab-arm exchange typically occurs over hours to days, it is possible that IgG4 produced locally in large quantities can rapidly form immune complexes prior to this process. Immune complex formation would additionally be favored in the presence of a high concentration of allergen, as is likely the case in EoE triggered by cow’s milk allergens. The impaired epithelial barrier in the gut of EoE patients allows the entry of high quantities of allergens into deeper tissue [79]. The role of IgG4 in the pathogenesis of EoE is important and warrants further investigation. It should be noted that in most cases, high IgG4 titers contribute to allergic tolerance rather than disease.

A recent study showed the pathophysiological characteristics of IgG4-positive cells in sinonasal tissues in chronic rhinosinusitis, especially in eosinophilic chronic rhinosinusitis. The number of IgG4-positive cells was significantly higher in nasal polyps, especially those from severe eosinophilic chronic rhinosinusitis patients, than in uncinate tissues. IgG4 was mainly expressed in infiltrating plasma and plasmacytoid cells, and the number of IgG4-positive cells significantly and positively correlated with blood and tissue eosinophilia, radiological severity, serum total IgE levels, and poor postoperative outcome [15]. A recent clinical study showed that serum IgG4 levels were significantly higher in patients with moderate to severe eosinophilic chronic rhinosinusitis (ECRS) compared to those with no or mild ECRS. IgG4 levels were also significantly higher in asthmatic patients and in patients experiencing recurrence after surgery compared to controls, indicating that the serum IgG4 levels are indicative of disease severity and postoperative course in CRS [16]. Additionally, another clinical study showed that serum IgG4/IgE levels were elevated in CRSwNP patients compared to controls. On the other hand, elevated serum IgG4 levels were more strongly associated with asthma and tissue eosinophilia compared to IgE levels [80]. An observational study showed that asthma patients with elevated IgG4 levels have significantly higher blood eosinophilia, total IgE, and FeNO [81].

Furthermore, it was reported that elevated circulating IgE and IgG4 anti-Staphylococcus aureus enterotoxin B antibody levels and reduced IgA and IgM levels were detected in patients with atopic dermatitis compared to control individuals [82]. A significant correlation was found between positive respiratory syncytial virus IgG4 antibodies at year one and atopic dermatitis, as well as food allergy development. Using a binary logistic regression model, children with positive respiratory syncytial virus IgG4 antibodies were found to have a 2.73-fold increased risk of high positive total and/or allergen-specific IgE during the first two years of life [83].

Tissue IgE and IgG4 levels were found to be elevated in aspirin-exacerbated respiratory disease (AERD) compared to the controls. Single-cell RNA sequencing also revealed increased IL5RA, IGHG4, and IGHE in antibody-expressing cells from patients with AERD compared with antibody-expressing cells from patients with CRSwNP [84]. However, a recent study provided a new perspective on IgG4 and the importance of allergen components in asthma. In this study, it was found that high exposure to particular allergens in household dust promotes the production of both sIgE and sIgG4 antibodies. In contrast, allergens with lower abundance in dust are capable of inducing sIgE with minimal or no sIgG4. Consequently, less abundant allergens such as Fel d 4, Fel d 7, Der p 2, and Der p 23 might be more closely associated with asthma than expected because they stimulate less sIgG4 production [85]. The process of class switching to IgG4, similar to the generation of IgE, is regulated by the cytokines IL-4 and IL-13 and requires co-stimulation by CD40 [34]. Furthermore, prolonged antigenic stimulation and IL-4-signaling along the T helper 2-axis has been linked to IgG4 skewing, which was profoundly reduced in dupilumab-treated patients, as well as TNFi-treated patients, indicating a critical role for IL-4/IL-13 as well as TNF in vivo IgG4 class-switching [86,87]. However, in patients with severe refractory atopic dermatitis, an increase in allergen-specific IgG4 levels was observed following the combined administration of dupilumab and AIT [88]. Furthermore, a multicenter clinical study demonstrated that there was no alteration in the peanut-specific IgG4 level after dupilumab monotherapy [89]. Importantly, IL-10 might be directly involved in regulating class switch recombination toward IgG4, as it has been shown to reduce IgE secretion and increase IgG4 production [34]. Recently, a specialized subset of follicular T helper (Tfh) cells has been identified in the tertiary lymphoid tissues of patients suffering from IgG4-related disease. These cells express markers such as BCL6, CXCR5, and ICOS as well as cytokines IL-4, IL-21, and IL-10, which might crucially regulate the class switching to IgG4 [90].

### 5.2. Pathogenic Role of IgG4 in Autoimmune Diseases

Autoimmunity refers to the lack of tolerance to self-antigens and can arise through various mechanisms. The recognition of self-antigens is a fundamental property of a healthy immune system, and its dysfunction is the basis of autoimmune diseases. Tolerance involves both central and peripheral mechanisms that require the correct functions of T and B cells [91].

In recent years, IgG4 has been detected in various autoimmune diseases, including neurological, dermatologic, hematologic, and rheumatologic diseases [23]. Due to its properties, such as the lack of complement activation and weak binding to Fc receptors to prevent trigger activation, the role of IgG4 in autoimmune diseases was unexpected [92]. Of note, Fc-independent mechanisms have been proposed as key players in IgG4-related autoimmunity. These include blocking protein–protein interactions and direct or indirect activation of enzymes or receptors by competitive or allosteric binding [23]. A poorly understood aspect of IgG4 is its ability to mimic the activity of the IgG rheumatoid factor by interacting with IgG on a solid support. Unlike conventional RF, the binding activity of IgG4 is located in its constant domains [92], creating a potential source of false positives in IgG4 antibody assays [1].

Koneczny I et al. proposed a classification system to determine the pathogenicity of IgG4 in IgG4-related autoimmune diseases (IgG4-AIDs) based on the following criteria: (1) the presence of IgG4 autoantibodies specific to the extracellular antigen in the affected organ, (2) in vitro detection of the pathogenicity of these autoantibodies, (3) reproducibility of the disease in experimental animal models through the passive transfer of patient sera with purified antibodies [23]. Based on the fulfillment of these criteria, IgG4-AID was categorized into three classes. Class I diseases provide the most evidence of IgG4-related disease. Examples of class I diseases include myasthenia gravis (targeting muscle-specific kinase antigen), chronic inflammatory demyelinating polyradiculoneuropathy (targeting contactin-1 and neurofascin155 antigens), thrombotic thrombocytopenic purpura (targeting a disintegrin and metalloproteinase with thrombospondin motifs 13 (ADAMTS13) antigen), and pemphigus (targeting the desmoglein 1 and desmoglein 3 antigens). The pathogenic role of IgG4 antibodies in class II diseases is shown in in vitro experiments. Within this group, limbic encephalitis (antigens: LGI1, CASP2R), GPIHBP1 autoantibody syndrome (antigen: glycosylphosphatidylinositol-anchored high-density lipoprotein-binding protein 1), membranous nephropathy (antigens: PLA2R, THSD7A), and mucous membrane pemphigoid (antigen: laminin 332) have been described [12,18]. The evidence is less strong in class III diseases due to the presence of Fc-dependent mechanisms, including chronic inflammatory demyelinating polyradiculoneuropathy, wherein the antibodies are driven against CASPR1, NF 140, NF 186, membranous nephropathy (antigens; alfa-enolase, superoxide dismutase 2 (SOD2), aldose reductase), anti-p200 pemphigoid (antigen; P200), Good pasture`s syndrome (antigen; type 4 collagen), ANCA associated vasculitis (antigens; antineutrophil cytoplasmic antibodies (ANCA)), bullous pemphigoid (antigens; BP 180 and BP 230), encephalitis (antigen; dipeptidyl-peptidase-like protein 6 (DPPX)), IgLON5 parasomnia (antigen; IGLON family member 5 (IgLON5)), and autoimmune polyendocrinopathy-candidiasis-ectodermal dystrophy (APECED) (antigens; IFN 1, IL-17A, IL-22) [12,18].

IgG4-related disease (IgG4–RD) represents a chronic, relapsing, immune-mediated fibroinflammatory disorder. In this condition, IgG4 antibodies are detectable at the affected anatomical sites. For a diagnosis of IgG4–RD, elevated serum IgG4 levels or an augmented number of IgG4+ plasma cells in tissue need to be accompanied by relevant clinical, histopathological, and frequently, radiological data [93]. In IgG4-RD, the massive expansion of polyclonal non-malignant IgG4-switched B cells and/or plasma cells leads to organ dysfunction [14]. IgG4-RD has been characterized by an initial inflammatory phase followed by a fibrotic process. It has been proposed that the interaction between B cells and CD4+ cytotoxic T cells (CTLs) drives these CTLs to induce cytokines, such as IL-1β, INF-γ, and TGF-β, responsible for fibrosis. These cells can be reduced with the anti-CD20 chimeric antibody rituximab [94]. However, research has shown that seronegative IgG4–RD patients exhibit lower serum IgG levels, eosinophil counts, IgE levels, and IgG4–RD responder index scores, along with a smaller number of affected organs compared to other subgroups [95]. Additionally, normal serum IgG4 levels have been observed in IgG4-related hypophysitis [96]. The main difference between IgG4-RD and IgG4-AID lies in the following: IgG4–RD is a well-defined fibro-inflammatory disease. In contrast, IgG4–AID encompasses a diverse range of disorders where IgG4 autoantibodies can be detected, but the underlying disease mechanisms are highly heterogeneous.

The antigen specificity of IgG4 in IgG4–RD is currently under intense investigation, and several findings have already emerged. In a cohort of IgG4–RD patients, the authors employed immunoaffinity chromatography and mass spectrometry on plasmablast clones. The findings revealed that a specific proportion (28%) of patients possessed IgG4-specific antibodies directed against galectin-3. This indicates that galectin-3 could potentially be one of the target antigens in IgG4–RD, yet the precise part it plays in the disease’s pathogenesis demands further investigation [97]. Additionally, trypsinogen has been recognized as a potential antigen in IgG4-related pancreatitis. Histopathological investigations have shown that in patients with chronic IgG4-related pancreatitis, the number of acinar cells experiences a significant decline. The existence of antibodies against trypsinogen was reproduced in the animal models of this disease, thereby validating this discovery [98].

It has been suggested that IgG4 can activate the alternative complement pathway, but there is still some controversy. Membranous nephropathy is an immune kidney disease with glomerular subepithelial immune complexes (ICs) of antigen, IgG, and complement activation products. Proteinuria results from complement-mediated podocyte injury. But complement activation pathways are debated, as IgG4, the dominant IgG subclass in ICs, was thought to be unable to activate complement. THSD7A, a transmembrane protein expressed on podocytes, is the target autoantigen in ~3% of cases of primary membranous nephropathy. A recent study implied that THSD7A ICs, predominantly containing IgG4, activate the complement at high IgG4 densities. This activation strictly requires a functional alternative pathway, whereas the classical and lectin pathways are dispensable [99]. Furthermore, another study showed that IgG4 autoantibodies can attenuate systemic lupus erythematosus progression by suppressing complement consumption and inflammatory cytokine production [100]. Additionally, a recent investigation has put forward contrasting viewpoints. In membranous nephropathy, hypoglycosylated anti-M-type phospholipase A2 receptor (PLA2R) IgG4 targets PLA2R on the surface of podocytes and activates the lectin complement pathway through mannose-binding lectin, rather than the alternative complement pathway. [101].

### 5.3. Role of IgG4 in Chronic Infection Disease

While the tolerance and immune suppressive properties of IgG4 in allergy immunotherapy are a blessing, they can also be detrimental in long-term and/or chronic infections [12]. As IgE is crucial for clearing helminth infections, a balanced Th1/Th2 immune response is required [12,102]. A chronic infection with a Th2 profile, however, skews the antibody ratios toward IgG4 [102].

Helminths can facilitate this Th2 profile through the induction of IL-10. *Schistosoma mansoni* soluble egg antigens induce IL-10 production in B cells upon internalization [103,104]. Other helminths manipulate macrophages and dendritic cells to produce IL-10 and other anti-inflammatory mediators [105,106]. Some types of nematodes also evade immune responses through the induction of T regulatory cells [10]. This weakens the efficacy of the IgE response, facilitating immune evasion by the parasites and, therefore, prolonging infection [4,102]. The ratio of parasite-specific IgE to IgG4 antibodies also correlates with re-infection rates in A. Lumbricoides cases; higher IgE levels have shown significant protection [107]. Elevated IgG4 levels have correlated with higher prevalence and higher parasite burden in *S. mansoni* infections [108]. They are also characteristic of asymptomatic helminth infections, such as lymphatic filariasis [4,109]. In the latter case, however, the suppression of the immune reaction prevents the infection from becoming pathologic (elephantiasis) as the death of the parasites and their larvae releases the endosymbiotic *Wolbachia* bacteria. The strong immune response to the bacterial antigens drives the pathology [4,10].

IgG4 plays a dual role in viral infections such as SARS-CoV-2 by conferring protection and prolonging infection. Higher than normal IgG4 serum levels were observed in relation to more severe SARS-CoV-2 cases [110,111]. Studies analyzing serum IgG levels from hospitalized patients showed a significantly higher mortality rate if IgG4 serum levels were elevated during the infection [111]. These findings suggest that IgG4 production induced by T regulatory cells aggravates the severity of COVID-19 [112]. The SARS-CoV-2 virus can induce IL-6 production by its open reading frame 8 (ORF8) protein, which in turn promotes the proliferation of T cells [113,114]. IgG4 antibodies against the virus spike protein have a lower potency for inducing antibody-mediated phagocytosis and compete with IgG1 for epitopes. This situation allows the virus to escape the immune responses and potentially prolong the infection [110,115]. However, the causality between severe infections causing high IgG4 levels and being the result of them is unclear.

Recent studies have also shown that mRNA vaccines induce high IgG4 levels specific to SARS-CoV-2 antigens [115,116]. A shift toward spike-specific IgG4 has been observed with additional booster vaccinations [86,115,116]. As these spike-specific IgG4 levels still had high avidity, the non-inflammatory properties could prevent immunopathology while still giving robust protection against the virus [115,117]. As such, this raises the question of whether IgG4 is protective or pathogenic in COVID-19 and warrants further investigation.

### 5.4. Role of IgG4 in Neoplasms

IgG4 titers are known to increase in response to chronic inflammation and persistent antigen stimulation, promoting immune tolerance to avoid excessive immune damage [118,119]. As persistent antigen stimulation is also common in neoplastic conditions, it is not surprising that IgG4, in relation to its described immune regulatory functions, has been associated with tumor progression [17,19,120,121,122,123]. The microenvironment within many tumors is skewed toward a Th2/Treg-milieu, where the release of IL-4 and IL-10 polarizes tumor-infiltrating B cells toward IgG4 isotype switching [19,112,124,125,126,127,128]. Nevertheless, the precise role of IgG4 and IgG4-positive B cells in malignancies is largely unknown, although several mechanisms on the role of IgG4 in tumor immune evasion have been suggested, including the following: (1) IgG4 neutralizes tumoricidal IgG1 via the Fc–Fc linkage (e.g., colon and breast cancer) [17,19,122,129], a process shown augmented by glutathione [130], (2) IgG4 blocks tumor specific binding sites and the IgG1–FcγR linkage, thus inhibiting subsequent immune activation and tumoricidal phagocytosis by pro-inflammatory macrophages [5,17,19,122,123,129] (e.g., melanoma), (3) IgG4 inhibits IgG1’s capacity to form antigen cross-linkage and immune complex formation through IgG1-IgG4 Fab-arm exchange (e.g., esophageal cancer) [17,19,122,129], (4) IgG4 promotes macrophage differentiation toward an anti-inflammatory M2b phenotype through FcγR binding [17,131,132], 5) a subset of IgG4-positive B cells produce pro-angiogenic cytokines that enhance tumor metastasis (e.g., melanoma) [133].

IgG4 has specifically been described as a pathologic constituent in melanoma [5,133,134,135,136], lung adenocarcinomas [130,137], thyroid cancer [138,139], esophageal cancer [130], gastric cancer [140], pancreatic cancer [127,141,142,143,144], liver cancer [145], extrahepatic cholangiocarcinoma [146,147,148], intrahepatic cholangiocarcinoma [149], colon cancer [129,130,131,150], and glioblastoma [128]. Furthermore, increased IgG4 serum levels and/or increased infiltrates of IgG4-positive plasma cells have been suggested as potential risk predictors of the development or disease relapse of melanoma [5,19], gastric cancer [140], liver cancer [145], extrahepatic cholangiocarcinoma [146], and intrahepatic cholangiocarcinoma [149]. However, using IgG4 as a diagnostic tool must be done with caution, as several reports have highlighted that serum IgG4 levels increase in non-carcinogenic autoimmune conditions where so-called pseudo-tumors also develop [151,152,153,154,155,156,157,158,159,160]. Thus, serum IgG4 and tumor-like tissue formation alone should not be diagnostically decisive without analyzing a biopsy of the targeted tissue or other additional diagnostic approaches, as demonstrated in pancreatic cancer patients with elevated IgG4 serum levels [161,162].

## 6. Conclusions

IgG4 plays a critical role in modulating allergic responses and promoting natural tolerance to allergens through unique mechanisms. The induction of IgG4 is heavily reliant on IL-10, as evidenced by its association with high-dose antigen exposure models, acquired natural tolerance, and conditions such as bee venom tolerance and helminth infections. These scenarios highlight the dual role of IgG4, namely, mitigating excessive type 2 responses while contributing to infection persistence or allergen tolerance. Furthermore, elevated IgG4 levels are observed in various contexts, from prolonged exposure to animal allergens to the natural resolution of food allergies, such as cow’s milk allergy. In conclusion, IgG4 antibodies are a double-edged sword in human health and disease. In health, they contribute to immune tolerance and homeostasis via Fab-arm exchange, preventing overactive immune responses. However, in diseases, their role is highly complex. In IgG4-related diseases, abnormal IgG4 elevation leads to tissue damage and fibrosis, yet our understanding of the precise mechanisms remains incomplete. In infectious diseases, while they can signify past exposure, they also complicate antibody-based diagnostics. In cancer, IgG4 is potentially involved in tumor immune evasion.

Despite these insights, the precise mechanisms driving IgG4-mediated tolerance, particularly in different allergenic contexts, remain an area for further investigation. Understanding these pathways could offer new perspectives on managing allergic diseases and promoting tolerance. For IgG4-related diseases, the focus should be on developing targeted therapies to correct the dysregulated IgG4 response. Understanding the regulatory factors of IgG4 in different diseases will uncover novel therapeutic targets.

In summary, research on IgG4 antibodies is still evolving. Future studies need to clarify their roles comprehensively. By doing so, we can expect to translate this knowledge into innovative diagnostic and therapeutic approaches, ultimately enhancing human health and disease management.

## Figures and Tables

**Table 1:** The features and roles of IgG subclasses.

Characteristic	IgG1	IgG2	IgG3	IgG4	Refs.
Species	Human, mouse	Human, mouse	Human, mouse	Human	[21,22,23]
% of antibodies within the IgG class	Most (~60–70%)	Moderate (~20–30%)	Low (~5%)	Lowest (<5%)	[7,19,21,24]
Molecular mass (kD)	146	146	170	146	[19,24]
Hinge region	15 aa	12 aa	62 aa	12 aa	[24]
Fab-arm exchange	No	No	No	Yes	[7,19]
Complement activation	High	Moderate	Very high	Low	[25]
Fcγ receptor binding	Very high	Moderate	High	Low	[7,21,24]
Antibody (Ab)-mediated phagocytosis	High	Low	Very high	Low	[19,24,26]
Ab-mediated cellular cytotoxicity	High	Low	Very high	Low	[19,24,26]
Anti-inflammatory role	Limited	Low	Limited	Prominent	[7,12]
Dominance in allergies	Rare	None	Rare	Common	[7,27]
Role in autoimmunity	Common	Rare	Limited	Often associated with tolerance	[7,12]

## Data Availability

Not applicable.
